# QuickStats

**Published:** 2015-02-20

**Authors:** FROM THE NATIONAL CENTER FOR HEALTH STATISTICS

**Figure:**
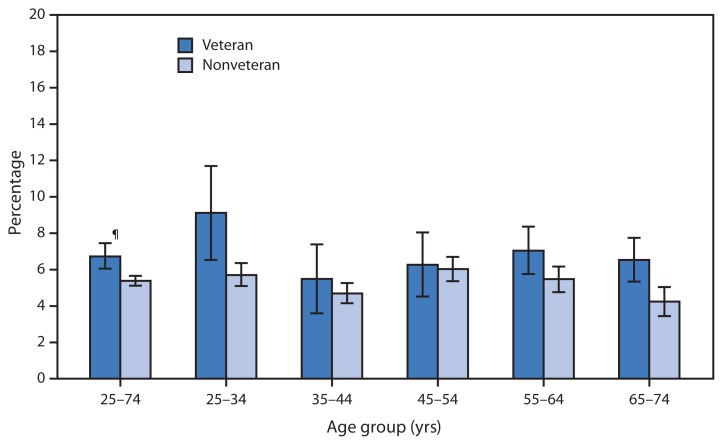
Percentage of Men Aged 25–74 Years Who Consume ≥15 Alcoholic Drinks Per Week,* by Age Group and Veteran Status^†^ — National Health Interview Survey, United States, 2011–2013^§^ *Defined as having consumed at least 12 alcoholic drinks (of any type) in lifetime, and in the last year consuming on average =15 alcoholic drinks per week. ^†^Veterans are those who have ever served on active duty in the U.S. Armed Forces, military reserves, or National Guard but are not now on full-time active duty with the armed forces. During 2011–2013, veterans accounted for 18% of the male population aged 25–74 years, ranging from 6% among men aged 25–34 years to 45% for those aged 65–74 years. ^§^Estimates are based on household interviews of a sample of the noninstitutionalized U.S. civilian population and are derived from the National Health Interview Survey sample adult component. ^¶^95% confidence interval.

During 2011–2013, male veterans aged 25–74 years were more likely to consume an average of ≥15 alcoholic drinks per week in the last year (“heavy drinking”) compared with nonveterans (7% versus 5%). Among men aged 25–34 years, the proportion of veterans who were heavy drinkers was 9%, higher than the 6% observed in nonveterans. Similarly, veterans were more likely than nonveterans to be heavy drinkers among men aged 55–64 years (7% versus 5%) and men aged 65–74 years (7% versus 4%). There was no significant difference in the proportion of veterans compared with nonveterans who were heavy drinkers among men aged 35–44 years or men aged 45–54 years.

**Source:** National Health Interview Survey data, 2011–2013. Available at http://www.cdc.gov/nchs/nhis.htm.

**Reported by:** Carla E. Zelaya, PhD, vdn3@cdc.gov, 301-458-4164; Renee M. Gindi, PhD.

